# Perspectives of Patients with Insulin-Treated Type 1 and Type 2 Diabetes on Hypoglycemia: Results of the HAT Observational Study in Central and Eastern European Countries

**DOI:** 10.1007/s13300-018-0388-2

**Published:** 2018-03-09

**Authors:** Martin Haluzik, Adam Kretowski, Krzysztof Strojek, Leszek Czupryniak, Andrej Janez, Peter Kempler, Michal Andel, Tsvetalina Tankova, Mihail Boyanov, Lea Smircic Duvnjak, Laszlo Madacsy, Iwona Tarnowska, Marcin Zychma, Nebojsa Lalic

**Affiliations:** 1 Diabetes Centre and Centre for Experimental Medicine, Institute for Clinical and Experimental Medicine, Vídeňská 1958/9, 140 21 Prague 4, Czech Republic; 2 Department of Endocrinology, Diabetology and Internal Diseases, Medical University of Białystok, Białystok, Poland; 3 Department of Internal Diseases Diabetology and Cardiometabolic Diseases, School of Medicine with the Division of Dentistry (SMDZ) in Zabrze, Medical University of Silesia, Katowice, Poland; 4 Department of Diabetology and Internal Medicine, Medical University of Warsaw, Warsaw, Poland; 5 Department of Endocrinology, Diabetes and Metabolic Diseases, University Medical Center, Ljubljana, Slovenia; 6 First Department of Medicine, Faculty of Medicine, Semmelweis University, Budapest, Hungary; 7 Center for Research of Nutrition, Metabolism and Diabetes, Third Faculty of Medicine, Charles University, Prague, Czech Republic; 8 Clinical Center of Endocrinology, Medical University–Sofia, Sofia, Bulgaria; 9 Clinic of Endocrinology and Metabolism, Department of Internal Medicine, University Hospital Alexandrovska, Medical University–Sofia, Sofia, Bulgaria; 10 Vuk Vrhovac University Clinic for Diabetes–UH Merkur, School of Medicine, University of Zagreb, Zagreb, Croatia; 11 First Department of Pediatrics, Faculty of Medicine, Semmelweis University Budapest, Budapest, Hungary; 12 Novo Nordisk Pharma, Warsaw, Poland; 13 Clinic for Endocrinology Diabetes and Metabolic Diseases, Clinical Center of Serbia (CCS), Faculty of Medicine, University of Belgrade, Belgrade, Serbia

**Keywords:** Diabetes, Healthcare costs, Hypoglycemia, Hypoglycemia fear, Insulin therapy

## Abstract

**Introduction:**

The aim of this study was to determine the level of awareness of hypoglycemia, the level of fear for hypoglycemia, and the response to hypoglycemic events among insulin-treated diabetes patients from Central and Eastern Europe (CEE). The impact of hypoglycemia on the use of healthcare resources and patient productivity was also assessed.

**Methods:**

This was a multicenter, non-interventional, two-part, patient self-reported questionnaire study that comprised both a retrospective cross-sectional evaluation and a prospective observational evaluation. Study participants were insulin-treated adult patients with type 1 diabetes mellitus (T1DM) or type 2 diabetes mellitus (T2DM) from CEE.

**Results:**

Most patients (85.4% T1DM and 83.6% T2DM) reported normal hypoglycemia awareness. The median hypoglycemia fear score was 5 out of 10 for T1DM and 4 out of 10 for T2DM patients. Patients increased glucose monitoring, consulted a doctor/nurse, and/or reduced the insulin dose in response to hypoglycemia. As a consequence of hypoglycemia, patients took leave from work/studies or arrived late and/or left early. Hospitalization was required for 31 (1.2%) patients with T1DM and 66 (2.1%) patients with T2DM.

**Conclusion:**

Hypoglycemia impacts patients’ personal and social functioning, reduces productivity, and results in additional costs, both direct (related to increased use of healthcare resources) and indirect (related to absenteeism.

**Funding:**

Novo Nordisk.

## Introduction

One of the most substantial risks related to the treatment of diabetes mellitus (DM) is hypoglycemia, which negatively affects patient’s health and overall quality of life (QoL) [1]. The results of the ACCORD clinical trial showed that intensive glucose-lowering therapy (with a target level of glycated hemoglobin [HbA1c] of < 6.0%) was associated with more frequent hypoglycemic events requiring medical or non-medical assistance and with higher mortality than less intensive treatment regimens [2].

Among the many negative long-term consequences of hypoglycemia, the higher risk of micro- and macrovascular events with potential fatal outcome is an important clinical concern as such events may occur months or years after episodes of severe hypoglycemia [3]. For example, hypoglycemia has been shown to cause cardiovascular (CV) events by increasing inflammation, endothelial dysfunction, and abnormal sympathoadrenal responses and by activating blood coagulation [4]. Novel drugs associated with lower risk of hypoglycemia (either in monotherapy or as an addition to standard antidiabetic therapy) can, however, reduce the CV risk in T2DM patients [5, 6]. Hypoglycemia can also affect cognitive function. For example, a history of severe hypoglycemic events has been associated with dementia [7]. Recurrent hypoglycemia has also been shown to cause chronic mood disorders, including depression and anxiety [8]. In addition, hypoglycemia has been shown to impair personal and social functioning and reduce QoL in patients with type 1 diabetes (T1DM) and type 2 diabetes (T2DM), resulting in problems related to employment, the ability to drive a motor vehicle, physical activity, and the fear of being dependent on other family members or caregivers. Indeed, this fear of dependency on caregivers and loss of self-control has been shown to influence the interpersonal relationships of patients affected by hypoglycemia [9].

The American Diabetes Association (ADA) clinical recommendations, published in 2017, underline the essential role of patients in preventing and managing hypoglycemia [1]. A patient-centered approach not only includes close direct communication, but also patient-reported outcomes, structured and individual patient education, individualized treatment, and self-monitoring and self-management of the DM [10]. Indeed, patients’ understanding of the disease and its complications is a key factor to the successful management of any chronic disease, and DM with its risk of hypoglycemia is no exception.

Nonetheless, the striking results of an analysis based on online questionnaires revealed that 65% of patients with T1DM and 50–59% of patients with T2DM rarely or never informed their general practitioner/specialist of hypoglycemic events, while 16 and 26%, respectively, had not been asked about hypoglycemia during routine visits [11]. Some patients did not discuss hypoglycemia with their physicians thinking that it is a “private issue” or a “personal failure”; others did not understand the importance of hypoglycemia [12].

Recently published findings of the HAT study, a large global patient-reported study on hypoglycemia, indicated high rates of hypoglycemia, with large variations between geographical regions [13]. These observed regional differences in hypoglycemia incidence raised the question as to whether they had resulted from true ethnic and population variations, from differences in treatment modalities [14], or from differences in the ways patients perceive and manage their disease.

Therefore, the aim of this study was to analyze in detail the HAT data reported by T1DM and T2DM patients from Central and Eastern Europe (CEE) according the patient’s perspective, which was not presented in the paper by Khunti et al. [13]. We focused on patients’ perception of hypoglycemia, the impact of hypoglycemia on patients’ personal and societal functioning, and utilization of healthcare resources.

## Methods

### Study Design and Subjects

This was a multicenter, non-interventional, two-part study based on a patient self-assessment questionnaire (SAQ) comprising both a 6-month and a 4-week retrospective cross-sectional evaluation (Part 1) and a 4-week prospective observational evaluation (Part 2). The study was conducted between 5 September 2012 and 30 December 2013 at 262 sites in CEE. Consecutive eligible patients were enrolled in the study during a routinely scheduled clinical consultation with their healthcare provider. Participants of this study came from Bulgaria, Croatia, Czech Republic, Hungary, Poland, Romania, Serbia and Montenegro, Slovakia, and Slovenia.

Each country was to identify 80–150 investigators (doctors working in either primary or secondary care). Each investigator was to recruit and enroll five to ten consecutive patients who met the inclusion criteria, namely, adults with T1DM or T2DM treated with insulin for at least 12 months. Illiterate patients or patients otherwise unable to complete a written survey were excluded from the study. Patients who were hospitalized at the time of study start were also excluded.

### Assessments

Part 1 of the questionnaire was used to record baseline demographic and clinical information. A history of hypoglycemic events was established, with data on severe events collected for 6 months and 4 weeks, respectively, prior to baseline and data on non-severe events collected for only 4 weeks prior to baseline. Information on each patient’s knowledge, awareness, and fear of hypoglycemia was also collected. Part 1 of the questionnaire was completed during routine clinical consultations with healthcare providers.

Part 2 of the questionnaire was completed 4 weeks after baseline and evaluated the occurrence of both severe and non-severe hypoglycemic events over the 4 weeks following entry into the baseline study. To assist recall and maintain anonymity, patients were provided with a diary to record hypoglycemic events. If a patient recorded more hypoglycemic events using the patient diary than recorded in Part 2 of the SAQ, then the data in the patient diary were used to calculate the prevalence of hypoglycemia in the 4 weeks after baseline as a mean to compensate for potential underestimations attributable to recall bias. Part 2 of the questionnaire and the patient diary were returned by post.

Parts 1 and 2 of the questionnaire were both used to evaluate patients’ responses to hypoglycemic events and the effect of these events on healthcare utilization and productivity during the time frame of the study.

In accordance to the ADA definition, hypoglycemia was defined as an acute complication of DM with a ≤ 3.9 mmol/L (70 mg/dl) fall in blood glucose level that exposes a patient to potential harm [15, 16]. Non-severe hypoglycemia was defined as an event managed by the patient alone; severe hypoglycemia was defined (based on the ADA definition) as any hypoglycemic event requiring assistance of another person to administer carbohydrate, glucagon, or other resuscitative actions [16]. Nocturnal hypoglycemia was defined by protocol as any hypoglycemic event occurring between midnight and 0600 hours.

The awareness of hypoglycemia was categorized based on answers given to the question ‘Do you have symptoms when you have a low sugar level?’, where ‘always’ and ‘usually’ denoted normal, ‘occasionally’ denoted impaired, and ‘never’ denoted severely impaired awareness.

Fear of hypoglycemia was assessed by the patients themselves on 0 to 10 semi-quantitative scale, where ‘0’ denoted not afraid at all, and ‘10’ denoted absolutely terrified.

### Compliance with Ethics Guidelines

This study was conducted in accordance with the Declaration of Helsinki (2004) and the International Conference on Harmonization (ICH) Guidelines for Good Clinical Practice (1996). The study design was approved by the country-specific regulatory authorities and ethical committees. All participants of the study provided signed informed consent.

### Statistical Analysis

Baseline refers to data collected using the Part 1 SAQ, while follow-up refers to data collected using the Part 2 SAQ and, where applicable, the patient diary. Continuous data were summarized in terms of the number of observations, mean, standard deviation, upper quartile, median, lower quartile, minimum, maximum, and missing number of observations, unless otherwise stated. Categorical data were summarized in terms of the number of patients providing data at the relevant time point (*n*), frequency counts, and percentages. Incidence rates of hypoglycemia together with the 95% confidence intervals were calculated as the number of events per patient year. Statistical analysis was performed using SAS® version 9.2 or later (SAS Institute, Cary, NC, USA).

## Results

### Baseline Characteristics

In total, 10,414 patients were invited to participate in the study. Overall, 9504 patients completed Part 1 of the SAQ (3135 with T1DM and 6369 with T2DM); 9229 patients (3040 with T1DM and 6189 with T2DM) completed Part 2 of the SAQ, and 7647 patients (2826 with T1DM and 4821 with T2DM) completed patient diaries.

### Demographic and Clinical Characteristics

Disposition of the patients by country was as follows: 537 (5.6%) were from Bulgaria, 315 (3.3%) were from Croatia, 1472 (15.5%) were from the Czech Republic, 1667 (17.5%) were from Hungary, 2448 (25.8%) were from Poland, 1190 (12.5%) were from Romania, 1064 (11.2%) were from Serbia and Montenegro, 500 were from Slovakia (5.3%), and 311 were (3.3%) from Slovenia. Patients with T1DM were younger than those with T2DM and had a longer mean duration of insulin use. Mean HbA1c level was similar in both groups of patients. The last measured HbA1c value in > 50% of all patients was in the range of 53–75 mmol/mol (7.0–9.0%). Most patients were treated with short-acting and long-acting insulins, and about 25% of all patients took oral anti-diabetes drugs. Most patients in the overall population experienced hypoglycemic events. Table 1 provides detailed information on patient baseline characteristics.

**Table 1:** Demographic and clinical characteristics at baseline (study entry)

Demographic and clinical characteristics	T1DM patients (*N* = 3135)	T2DM patients (*N* = 6369)
Age (years)		
Mean (SD)	42.0 (13.97)^b^	62.5 (9.30)^b^
Sex [*n* (%)]		
Male	1511 (48.3)^b^	3095 (48.7)^b^
Female	1618 (51.7)	3265 (51.3)
Employment status [*n* (%)]		
Student	234 (7.5)	3 (< 0.1)
Full-time employment	1539 (49.5)	1140 (18.1)
Part-time employment	244 (7.8)	327 (5.2)
Unemployed	272 (8.7)	296 (4.7)
Pensioned	689 (22.1)	4427 (70.3)
Other	133 (4.3)	107 (1.7)
Duration of diabetes (years)		
*n*	3130	6331
Mean (SD)	16.8 (11.17)^b^	13.6 (7.87**)**^b^
Duration of insulin treatment (years)		
*n*	3132	6351
Mean (SD)	16.3 (11.17)^b^	7.1 (5.78)^b^
Self-reported last HbA1c (mmol/mol) levels		
*n*	2879	5438
Mean (SD)	61.0 (15.68)^b^	60.9 (15.02)^b^
Self-reported last HbA1c (%) levels^a^		
Mean (SD)	7.7 (3.6)	7.7 (3.5)
Patients with self-reported last HbA1c (%) levels [*n* (%)]		
*n*	2879	5438
< 7.0%,	905 (31.4)	1549 (28.5)
≥ 7.0% and ≤ 9.0%	1513 (52.6)	3147 (57.9)
> 9.0%	461 (16.0)	742 (13.6)
Treatment of diabetes [*n* (%)]		
Short-acting insulin	2718 (86.9)	3451 (54.6)
Long-acting insulin	2525 (80.7)	3895 (61.6)
Mixed insulin	166 (5.3)	2381 (37.6)
Insulin pump	416 (13.3)	53 (0.8)
Oral anti-diabetes treatments	84 (2.7)	2124 (33.6)
Injectable anti-diabetes treatments (excluding insulin)	3 (< 0.1)	51 (0.8)
Use of continuous glucose monitoring device [*n* (%)]		
Yes	536 (17.4)	715 (11.5)
No	2486 (80.9)	5338 (85.9)
Not sure	52 (1.7)	160 (2.6)
Self-measurement of blood glucose levels [*n* (%)]		
Yes	3119 (99.6)	6303 (99.1)
No	12 (0.4)	58 (0.9)
Self-reported hypoglycemia [*n* (%)]		
Yes	3069 (98.3)	5357 (84.6)
No	44 (1.4)	829 (13.1)
Not sure	8 (0.3)	143 (2.3)

*HbA1c* Glycated hemoglobin;* SD* standard deviation,* T1DM* type 1 diabetes,* T2DM* type 2 diabetes

^a^Calculated

^b^Reported previously by Khunti et al. [13]

### Knowledge about hypoglycemia

At baseline, most of the patients were familiar with the definition of hypoglycemia, and most had experienced a hypoglycemic episode. More than 40% of patients recognized hypoglycemia on the basis of symptoms and low blood glucose concentration.

In total, a minority of patients who used blood glucose measurement to determine if they had hypoglycemia provided values inconsistent with the standard definition of a hypoglycemic event. These details are given in Table 2.

**Table 2:** Patient knowledge of hypoglycemia (baseline)

Patient knowledge of hypoglycemia at baseline	T1DM patients (*N* = 3135)	T2DM patients (*N* = 6369)
Knowledge of the definition of hypoglycemia [*n*/*N* total (%)]	3061/3121 (98.1)	5835/6330 (92.2)
Experienced hypoglycemia [*n*/*N* total (%)]	3069/3121 (98.3)	5357/6329 (84.6)
Self-recognition of hypoglycemia based on [*n*/*N* total (%)]:		
Symptoms only	874/3041 (28.7)	1786/5245 (34.1)
Low blood glucose only	117/3041 (3.8)	325/5245 (6.7)
Either symptoms or low blood glucose	618/3041 (20.3)	762/5245 (14.5)
Both symptoms and low blood glucose	1432/3041 (47.1)	2345/5245 (44.7)
Blood glucose measurement to determine if they have hypoglycemia but provided values inconsistent with standard definitions (≤ 3.9 mmol/L; 70 mg/dl) [*n*/*N* total (%)]	372/2875 (12.9)	932/4711 (19.8)
Blood glucose level below which patients considered was a hypoglycemic event (mmol/L) in patients providing values consistent with standard definition, mean (SD)	3.16 (0.550)	3.22 (0.534)
Blood glucose level below which patients considered was a hypoglycemic event (mg/dl) in patients providing values consistent with standard definition, mean (SD)^a^	56.9 (9.91)	58.0 (9.62)
Blood glucose level below which patients considered was a hypoglycemic event (mmol/L) in patients providing values inconsistent with standard definition, mean (SD)	4.34 (0.608)	4.47 (0.625)
Blood glucose level below which patients considered was a hypoglycemic event (mg/dl) in patients providing values inconsistent with standard definition, mean (SD)^a^	78.2 (10.95)	80.5 (11.26)

^a^Calculated

### Incidence, Awareness and Fear of Hypoglycemia

#### Incidence of Hypoglycemia

During the 4 weeks preceding baseline, most of patients reported at least one hypoglycemic event. During the 4 weeks after baseline, the percentages of patients who reported hypoglycemic events were similar to those in the 4 weeks prior to baseline (Table 3).

**Table 3:** Incidence of hypoglycemic events in the 4 weeks before and 4 weeks after baseline (by diabetes type)

Incidence of hypoglycemic events	T1DM patients: 4 weeks before baseline (*N* = 3052)	T1DM patients: 4 weeks after baseline (*N* = 3052)	T2DM patients: 4 weeks before baseline (*N* = 6218)	T2DM patients 4 weeks after baseline (*N* = 6218)
Number of patients with any hypoglycemic event [*n* (%)]	2560 (84.3)	2583 (85.0)^a^	3509 (57.0)	3312 (53.8)^a^
Incidence rate ratio (4 weeks after/4 weeks before)	1.55		1.19	
95% CI for incidence ratio	1.45, 1.65		1.11, 1.27	
Number of patients with a severe hypoglycemic event [*n* (%)]	365 (12.2)	395 (13.0)	408 (6.7)	469 (7.6)
Incidence rate ratio (4 weeks after/4 weeks before)	1.30		1.35	
95% CI for incidence ratio	1.07, 1.58		1.07, 1.70	
Number of patients with a nocturnal hypoglycemic event [*n* (%)]	1305 (43.8)	1142 (38.8)	1358 (22.7)	1024 (17.3)
Incidence rate ratio (4 weeks after/4 weeks before)	0.77		0.70	
95% CI for incidence ratio	0.69, 0.85		0.64, 0.77	
Number of patients with a non-severe hypoglycemic event [*n* (%)]	2490 (82.9)	2550 (84.5)	3420 (57.0)	3238 (53.7)
Incidence rate ratio (4 weeks after/4 weeks before)	1.56		1.16	
95% CI for incidence ratio	1.46, 1.67		1.09, 1.24	

*CI* Confidence interval

^a^Reported previously by Khunti et al. [13]

The incidence of patient-reported nocturnal hypoglycemic events was much higher in the T1DM patient population than in the T2DM patient population (Table 3).

#### Awareness of Hypoglycemia

Hypoglycemia awareness at baseline was similar in T1DM and T2DM patients. Most patients reported normal awareness (85.4% of T1DM and 83.6% of T2DM patients). Approximately 13% of all patients had impaired hypoglycemia awareness (13.3% of T1DM and 13.4% of T2DM patients). A minority had severely impaired hypoglycemia awareness (1.3% of T1DM and 3.1% of T2DM patients).

#### Fear of Hypoglycemia

**Fig. 1:**
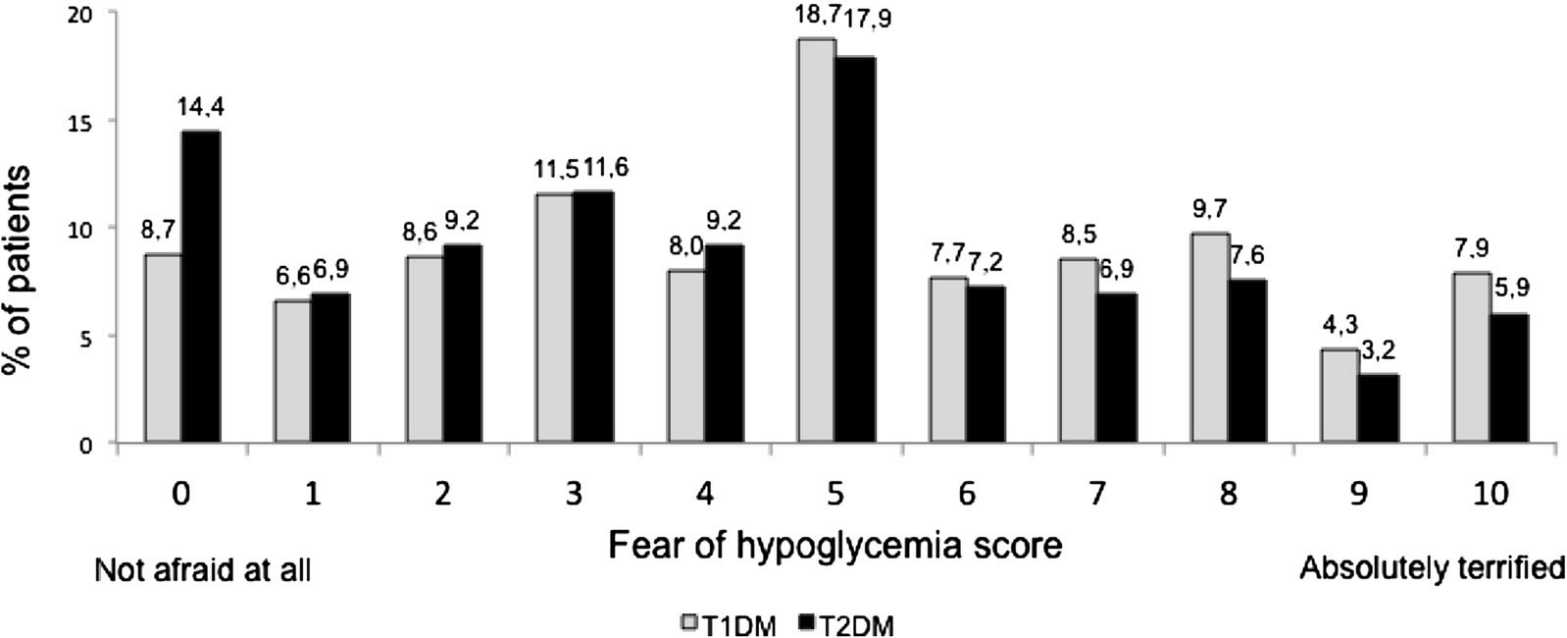
Fear of hypoglycemia by diabetes type at baseline.* T1DM* Type 1 diabetes mellitus,* T2DM* type 2 diabetes mellitus

At baseline, responses from T1DM patients indicated a greater fear of hypoglycemia than did those those of T2DM patients (Fig. 1).

### Patient Actions due to Hypoglycemia

A hypoglycemic event resulted in most patients increasing glucose self-monitoring, consulting a doctor or a nurse, and/or reducing the insulin dose (Table 4).

**Table 4:** Patient actions resulting from a hypoglycemic event 6 months before and 4 weeks after baseline by diabetes type

Patient actions	T1DM patients: 6 months before baseline (*N* = 2797)	T2DM patients: 6 months before baseline (*N* = 4129)	T1DM patients: 4 weeks after baseline (*N* = 2515)	T2DM patients: 4 weeks after baseline (*N* = 3140)
Consulted their doctor/nurse [*n* (%)]	1938 (70.7)	2960 (75.3)	1266 (53.0)	1822 (63.2)
Required any form of medical assistance [*n* (%)]	1954 (71.3)	3005 (76.5)	1280 (53.6)	1843 (63.9)
Increased carbohydrate intake (sugar or snacks) [*n* (%)]	1352 (50.5)	2080 (53.0)	1255 (52.3)	1544 (52.0)
Avoided physical exercise [*n* (%)]	585 (22.5)	910 (24.0)	499 (21.2)	694 (23.9)
Reduced insulin dose [*n* (%)]	1702 (63.5)	1720 (44.5)	1248 (51.7)	1205 (40.6)
Skipped insulin injections [*n* (%)]	354 (13.6)	423 (11.2)	199 (8.5)	202 (6.9)
Increased blood glucose self-monitoring [*n* (%)]	2059 (76.7)	2747 (71.0)	1831 (75.7)	2152 (71.9)

### Impact of Hypoglycemia on Healthcare Services—Direct Costs

The occurrence of hypoglycemia in the retrospective and prospective parts of the study led patients with T1DM to make additional telephone contact with a doctor or a nurse (15.5 and 9.2%, respectively) or to attend additional clinical appointments (7.1 and 3.0%, respectively). During the 6 months prior to baseline, 97 (3.5%) patients with T1DM had a hypoglycemic event requiring hospitalization, and during the 4 weeks after baseline, hypoglycemia occurred in 31 (1.2%) patients.

In the retrospective period, 652 (16.6%) patients with T2DM consulted medical professionals by telephone as a result a hypoglycemic event compared to 409 (14.2%) in the prospective period. Additional clinical appointments were made by 302 (7.7%) patients with T2DM in the 6 months preceding baseline and by 147 (5.1%) patients during the 4 weeks after baseline. In the 6 months preceding baseline, 141 (3.4%) patients with T2DM required hospitalization due to hypoglycemia compared to 66 (2.1%) during the 4 weeks after baseline.

### Impact of Hypoglycemia on Work and Study Attendance—Indirect Costs

Most patients with T1DM enrolled in the study were studying or working (2017 before baseline, 1957 in the 4 weeks after baseline). In the prospective period, 40 (2.5%) of these patients had taken some form of leave from work or studies (mean duration 2.3 ± 2.15 days), 68 (4.3%) had arrived late to their study or working place, and 64 (4.0%) had to leave early due to hypoglycemic events. In the retrospective period prior to baseline, 225 (11.4%) had taken leave (mean duration 4.7 ± 8.09 days), 316 (16.0%) had arrived late, and 309 (15.6%) had left work/studies early.

Among the 1431 patients with T2DM who were studying or employed in the 4-week prospective period, 19 (2.5%) had taken leave from work or studies (mean duration 2.8 ± 4.97 days), 20 (2.7%) had arrived late to their place of study or work, and 37 (4.9%) had to leave early due to hypoglycemic events. In the retrospective period prior to baseline, 74 (5.1%) had taken leave (mean duration 3.3 ± 3.09 days), 91 (6.4%) had arrived late, and 120 (8.4%) had left work/studies early.

## Discussion

The manner in which patients perceive hypoglycemia, how they handle hypoglycemic events and to what extent these complications affect patient actions and healthcare utilization were part of the HAT survey. Although these data were not presented for the total HAT population in a previous publication reporting the global HAT results [13], we consider such information to be important with respect to providing further insights into hypoglycemia prevention. Therefore, we undertook a detailed examination of these data for the CEE subpopulation.

We observed similar percentages of patients who reported hypoglycemic events in the retrospective and prospective phase of our study. Our interpretation is that once patients started the study, they became more compliant and thus their diabetes was better controlled, possibly including a lower actual rate of hypoglycemia.

Impaired hypoglycemia awareness is one of the strongest risk factors for severe hypoglycemia [17] and has been reported to increase the risk of severe hypoglycemia in T1DM patients by up to sixfold [18]. Data on the proportion of T1DM and T2DM patients unaware of hypoglycemia varies significantly among studies [11, 18–25]. In our study, most T1DM patients (85.4%) reported a normal level of hypoglycemia awareness, and only 13.3% of T1DM patients reported an impaired awareness.

The difference in the percentage of patients reporting impaired hypoglycemia awareness in this study as compared to previous research may be explained by the different ways the patient responses were classified. Most studies conducted to date have used a rigid classification proposed by Pedersen-Bjergaard et al. [26]. In our study, patients who answered the question ‘Do you have symptoms when you have a low sugar level?’ with ‘always’ or ‘usually’ were classified as having a normal level of hypoglycemia awareness, those who answered ‘occasionally’ were classified as impaired, and those who answered ‘never’ were classed as severely impaired. However, this classification of hypoglycemia awareness relies on patients’ subjective assessments and as such was not validated. Therefore, this methodology needs to be considered as one of the study limitations.

In our study, the fear of hypoglycemia was slightly higher in patients with T1DM than in patients with T2DM. However, most T1DM and T2DM patients scored their fear level as 5 in the 10-point scale (where 10 was ‘absolutely terrified’). Hypoglycemia fear influences glycemic variability, dietary patterns, and physical activity in patients with T1DM, as shown by Martyn-Nemeth et al. whose patients increased calorie intake and reduced physical activity due to their fear of hypoglycemia [27]. Other studies have found that fear of hypoglycemia is associated with diabetes-related QoL and psychological well-being [28, 29]. The fear of hypoglycemia is also a major reason for discontinuation of diabetes medications by T2DM patients [29, 30].

The occurrence of a hypoglycemic event resulted in most of the patients in our study increasing glucose self-monitoring and/or consulting a doctor or a nurse. Patients with T1DM were slightly more likely to increase the frequency of glucose self-monitoring compared to T2DM patients, but they were also more aware of hypoglycemic events. However, more than half of the T2DM patients decreased the insulin dose and some skipped insulin injections, which can in turn lead to poor glycemic control and increased risk of related health consequences, such as CV disorders.

The results of our study reflect patient reactions reported in previous studies. Fulcher et al. followed patient responses to nocturnal and daytime non-severe hypoglycemic events and reported that these patients decreased insulin dosage, contacted a healthcare professional, and performed additional blood testing in the week following the event [31]. Brod et al. also reported similar patient reactions to non-severe nocturnal hypoglycemia, namely, extra blood glucose level tests and reduced insulin dosage [32].

In our study we found that hypoglycemia resulted in an increased use of healthcare services and a decrease in work/study productivity—responses supported by previous research [31–33]. In the analysis by Fidler et al., hypoglycemia was associated with a reduction in QoL, increased fear and anxiety, reduced productivity, and increased healthcare costs [34]. Widz et al. estimated the average monthly direct cost of a hypoglycemic event to be approximately 700 EUR for severe events and 40 EUR for non-severe events [35]. In addition, Goldstein et al. found that severe hypoglycemic events increased the use of healthcare resources and healthcare costs during the month after the event as compared to the month before, with hospital admissions increasing by almost by 100% for T1DM patients and by 127% for T2DM patients, and the mean duration of hospitalization being longer [36]. The number of outpatient visits increased by 37% (T1DM) and 47% (T2DM). As a result, total monthly healthcare costs increased by 46% in T1DM patients and by 87% in T2DM patients [36].

An earlier analysis from seven European countries revealed that approximately 10% of both daytime and nocturnal non-severe hypoglycemic events led to mean work-time loss of about 1–3 h [33]. Annual costs of severe hypoglycemic events in nine European countries were estimated to be approximately 380,000 EUR in Macedonia ranging up to 58,430,000 EUR in Spain. When expressed as cost per drug-treated patient, the costs ranged from 5.5 EUR in Bulgaria to 17.7 EUR in Spain. The differences were attributable to the costs of a single event treatment and general differences in rates [37].

We also report how hypoglycemia affected patient absenteeism: patients arrived late at work/study, had to leave early, and 2.5% of them needed to take sick leave. Hypoglycemic events also led to an increase in healthcare services usage. Therefore, minimizing hypoglycemia risk while maintaining good glycemic control may reduce the overall costs of diabetes by diminishing direct costs related to the use of healthcare services and indirect costs by increasing work productivity.

The limitation of the HAT was its observational design with a longer retrospective time horizon and much shorter duration of the prospective phase. Therefore, the comparisons between the results from the retrospective and prospective phases should be interpreted with caution, mainly because of possible recall bias. For example, healthcare utilization or work or study attendance seem to be much lower during the retrospective phase than in the prospective phase, but this difference may be a consequence of underreporting, caused by a recall bias. Another limitation relates to the eligibility criterion that only patients attending routinely scheduled clinical consultations could be enrolled in the study. Illiterate patients or patients otherwise unable to complete a written survey, i.e. patients at the lowest cultural/socio-economic level were excluded from the study, possibly introducing a population bias. However, the study included a large patient population and allowed us to determine the real-life incidence of hypoglycemia as reported by patients. In addition, the HAT study enabled assessment of the impact of hypoglycemia on patients’ reactions, use of healthcare service, and work/study productivity. Another strong feature of the HAT study was that during the prospective period, patient diaries were used in addition to Part 2 of the SAQ to reduce recall bias. While the use of patient-reported data from the diaries in addition to Part 2 of the SAQ may have increased the reliability of the data pertaining to the prevalence of hypoglycemia, it has the potential to overestimate hypoglycemia rates.

It should be also highlighted that this analysis is entirely based on patient self-reported data. Patient-reported outcomes provide a clear picture of the patient’s perspective and perception. On the other hand, they are inevitably subjective and susceptible to imprecision and variation. Nevertheless, such self-assessments are inherent elements of a patient-centered healthcare system [38], which is particularly suited to diabetes where disease self-management is crucial for proper treatment.

## Conclusions

In conclusion, our study shows that hypoglycemia impacts patients’ personal and social functioning and leads to additional healthcare usage and loss of productivity. Based on these results, we suggest that there is a room for improving education relating to the way hypoglycemia is recognized by patients. We believe that the results of our study can be used to identify cost-effective solutions for improving blood glucose control and the QoL of patients with DM.
